# Olive Oil Phenols Prevent Mercury-Induced Phosphatidylserine Exposure and Morphological Changes in Human Erythrocytes Regardless of Their Different Scavenging Activity

**DOI:** 10.3390/ijms23105693

**Published:** 2022-05-19

**Authors:** Rosaria Notariale, Pasquale Perrone, Luigi Mele, Gennaro Lettieri, Marina Piscopo, Caterina Manna

**Affiliations:** 1 Department of Precision Medicine, School of Medicine, University of Campania “Luigi Vanvitelli”, 80138 Naples, Italy; notarialer@gmail.com (R.N.); pasqualeperrone1995@gmail.com (P.P.); 2 Department of Experimental Medicine, University of Campania “Luigi Vanvitelli”, 80138 Naples, Italy; luigi.mele86@gmail.com; 3 Department of Biology, University of Naples Federico II, 80126 Naples, Italy; gennarole@outlook.com (G.L.); marina.piscopo@unina.it (M.P.)

**Keywords:** calcium, endothelium, erythrocytes, glutathione, hydroxytyrosol, mercury, olive oil, oxidative stress, phosphatidylserine, tyrosol

## Abstract

Phosphatidylserine (PS) translocation to the external membrane leaflet represents a key mechanism in the pathophysiology of human erythrocytes (RBC) acting as an “eat me” signal for the removal of aged/stressed cells. Loss of physiological membrane asymmetry, however, can lead to adverse effects on the cardiovascular system, activating a prothrombotic activity. The data presented indicate that structurally related olive oil phenols prevent cell alterations induced in intact human RBC exposed to HgCl_2_ (5–40 µM) or Ca^2+^ ionophore (5 µM), as measured by hallmarks including PS exposure, reactive oxygen species generation, glutathione depletion and microvesicles formation. The protective effect is observed in a concentration range of 1–30 µM, hydroxytyrosol being the most effective; its in vivo metabolite homovanillic alcohol still retains the biological activity of its dietary precursor. Significant protection is also exerted by tyrosol, in spite of its weak scavenging activity, indicating that additional mechanisms are involved in the protective effect. When RBC alterations are mediated by an increase in intracellular calcium, the protective effect is observed at higher concentrations, indicating that the selected phenols mainly act on Ca^2+^-independent mechanisms, identified as protection of glutathione depletion. Our findings strengthen the nutritional relevance of olive oil bioactive compounds in the claimed health-promoting effects of the Mediterranean Diet.

## 1. Introduction

Erythrocytes (RBC) are highly deformable cells that can rapidly change their shape to fit through the blood vessels in the body. Moreover, their peculiar morphology of biconcave disc by increasing the surface/volume ratio facilitates gas exchanges, the main function of these highly specialized cells [1]. Besides the physiological role of oxygen and CO_2_ transport, these cells exert additional non-canonical functions, including regulation of nitric oxide (NO) bioavailability. Indeed, these cells express the eNOS isoform and show the ability to biosynthesize NO under oxygen deprivation and store it bound to specific cysteine residues of hemoglobin, contributing to cardiovascular homeostasis [2,3,4,5,6]. In mammals, mature cells come from a finally regulated process called erythropoiesis, involving loss of cell organelles. In addition, at the end of maturation, erythroblasts expel their nuclei, giving rise to reticulocytes released in the bloodstream and pyrenocytes, rapidly eliminated by macrophages, where phosphatidylserine (PS) exposure acts as an “eat me” signal [7,8]. Late enucleation of erythroblasts defines a starting point of an average aging process of 120 days until their elimination from circulation through recognition and phagocytosis mediated by tissue macrophages within the reticuloendothelial system in the spleen, liver (Kupffer cells), and bone marrow. Interestingly, this process is also associated with exposure of PS to the outer membrane leaflet. Thus, losing the physiological membrane asymmetric PS distribution turns out to be a key initiation signal in the erythrophagocytosis of aged RBC, thereby regulating the lifespan of circulating mature cells as well as the removal of damaged RBC in pathological situations [9,10,11]. In this respect, this membrane alteration is regarded as one of the mechanisms underlying the onset of anemia associated with chemotherapeutic treatments [12,13] as well as a broad range of diseases [14,15]. As a matter of fact, PS translocation to the cell surface represents a key hallmark of erythrocyte programmed cell death, also called eryptosis [16,17,18,19]. Similarly to apoptosis of nucleated cells, RBC can enter a programmed suicidal death that allows cell degradation without producing toxic by-products, preventing the release of harmful cellular components such as hemoglobin, heme and free iron that can favor plaque formation [20]. This specific membrane alteration, however, can lead to adverse effects on the cardiovascular system, by providing a binding site for prothrombinase complex as well as by inducing unusual adhesion between RBC with both endothelial cells and other cells in the bloodstream, thus resulting in clot formation and microvascular occlusion [21,22,23]. Increased microvesicles (MVs) formation has also recently been recognized as a contributing factor to thrombotic events [24,25].

Eryptosis is triggered by oxidative stress (OS) [26] as well as endogenous and exogenous substances, such as lysophosphatidic acid and heavy metal, respectively [27,28]. In this respect, our data and data in the literature show that RBC are particularly sensitive to mercury (Hg), acting as a preferential site for its accumulation [29,30]. In particular, Hg mainly binds to sulfhydryl groups of cellular thiols, mainly glutathione (GSH), thus impairing the endogenous defense system [31]. In addition, Hg rapidly reacts with accessible cysteine residues of both membrane as cytosolic proteins, resulting in severe metabolic and morphological alterations [30]. Interestingly, beside the well-known neuro and nephro toxic effects, this highly toxic wide-spread pollutant [32] can significantly affect cardiovascular health [33,34]. RBC have been proposed as contributing to endothelial dysfunction and thrombotic events [35].

Based on these observations, it would therefore be interesting to screen compounds able to reduce PS-related membrane modification. In this respect, recent data from our group showed that hydroxytyrosol (3,4-diidroxyphenylethanol, HT), an antioxidant phenol present in high concentrations in virgin olive oil, has the potential to inhibit PS exposure in human RBC, induced by different stressors, including Hg [36]. The aim of this study was to further evaluate the possible protective effects played by natural phenolic compounds in human RBC, particularly related to metabolic as well as morphological alterations associated with increased prothrombotic activity of these cells. In the search for an effective tool to counteract this damage, we performed a comparative analysis of the effects of HT and its natural olive monophenolic analogue tyrosol (Tyr) [37], as well as its in vivo metabolite homovanillic alcohol (HA) [38,39]. Intact human RBC have been subjected in vitro to treatment with mercuric chloride (HgCl_2_) or Ca^2+^-ionophore. It is well known, indeed, that alterations in calcium homeostasis are associated with Hg toxicity, and increased intracellular Ca^2+^ induces PS exposure [35]. The protective effect of the selected phenols and the underlying mechanism(s) were evaluated by measuring several hallmarks including PS exposure, reactive oxygen species (ROS) generation, GSH depletion and MVs formation. 

## 2. Results

**Figure 1:**
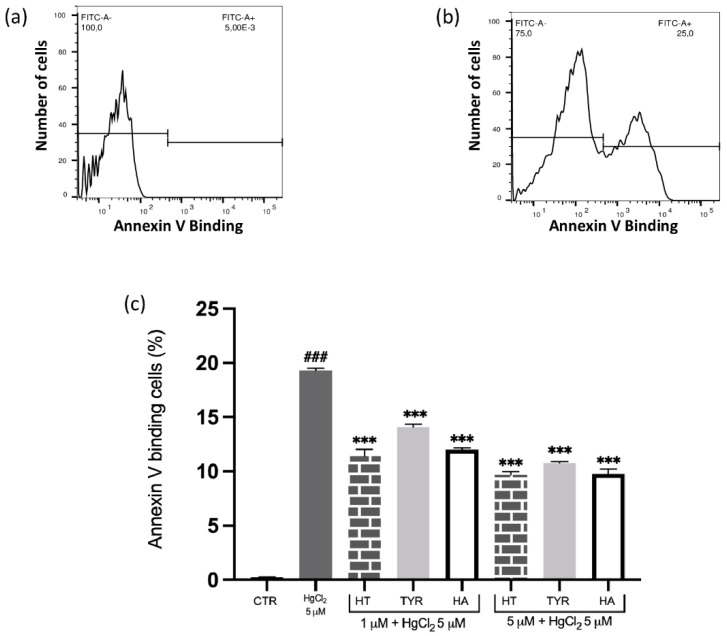
Effect of HT and its analogue on Hg-induced PS exposure in RBC. Cells were treated with HgCl_2_ in the presence of increasing concentrations of HT, Tyr and HA and PS exposure was evaluated as annexin-V-binding cells. (**a**) Original histogram of control cells (CTR). (**b**) Original histogram of Hg-treated cells. (**c**) Histogram of phenols effect on Hg treatment. Data are the means ± SD (n = 9). Statistical analysis was performed with one-way ANOVA followed by Tukey’s test. ### *(p* < 0.001) indicates a significant difference from CTR. *** *(p* < 0.001) indicates a significant difference from Hg treatment. PS: phosphatidylserine; RBC: red blood cell, HT: hydroxytyrosol; Tyr: tyrosol; HA: homovanillic alcohol.

To explore the ability of olive oil bioactive components in preventing Hg-induced cytotoxicity in human RBC and the underlying mechanism(s), several phenolic compounds, endowed with different antioxidant activities, were selected. Among them, HT and its in vivo metabolite HA show effective and comparable scavenging properties [39,40,41,42], while Tyr, which lacks the o-diphenolic structure, is characterized by a weak activity [43,44]. Intact RBC were exposed in vitro to HgCl_2_ and several hallmarks were evaluated, including PS translocation at the cell surface; PS-exposing RBC were identified utilizing annexin-V-binding, as determined by flow cytometry. As reported in Figure 1, and according to our previous findings, cell exposure to the heavy metal induces an increase of PS exposure. Under the same experimental conditions, treatment with all selected phenols results in a decrease of annexin-V-binding RBC in a dose-dependent manner, a significant protection occurring at a concentration as low as 1 µM. Still, Tyr appears to be less effective than HT and HA (Figure 1). 

**Figure 2:**
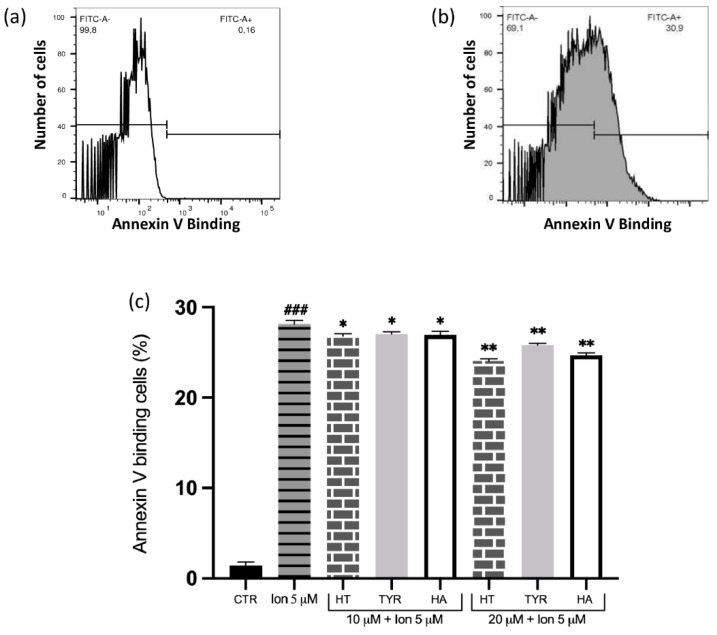
Effect of HT and its analogue on Ca^2+^-induced PS exposure in RBC. Cells were treated with Ca^2+^ ionophore in the presence of increasing concentrations of HT, Tyr and HA and PS exposure was evaluated as annexin-V-binding cells. (**a**) Original histogram of control cells (CTR). (**b**) Original histogram of Ca^2+^ ionophore-treated cells. (**c**) Histogram of phenols effect on Ca^2+^ ionophore treatment. Data are the means ± SD (n = 9). Statistical analysis was performed with one-way ANOVA followed by Tukey’s test. ### *(p* < 0.001) indicates a significant difference from CTR. ** *(p* < 0.01) and * *(p* < 0.05) indicates a significant difference from Ca^2+^ ionophore treatment. PS: phosphatidylserine; RBC: red blood cell, HT: hydroxytyrosol; Tyr: tyrosol; HA: homovanillic alcohol.

It is known that Hg toxicity is associated with alteration in calcium homeostasis and that increased intracellular Ca^2+^ induces PS exposure. We therefore investigated whether the tested phenolic compounds had a similar protective effect on Ca^2+^-induced PS exposure. As expected, cell incubation with 5 μM Ca^2+^ ionophore in the presence of 1 mM extracellular calcium significantly increases the percentage of annexin-V-binding RBC (Figure 2). Also in this case, in RBC treated with micromolar concentrations of the phenolic compounds, a significant reduction of PS exposing cells are observed, even though higher concentrations are required (10–20 µM).

**Figure 3:**
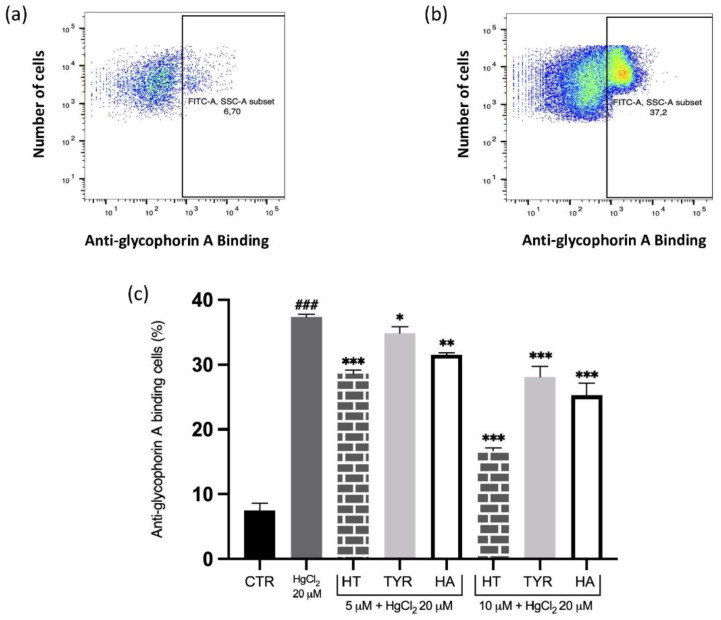
Effect of HT and its analogue on Hg-induced MVs formation from RBC. Cells were treated with HgCl_2_ in the presence of increasing concentrations of HT, Tyr and HA and MVs formation was evaluated using FITC anti-human glycophorin A antibody. (**a**) Original histogram of control cells (CTR). (**b**) Original histogram of Hg-treated cells. (**c**) Histogram of phenols effect on Hg treatment. Data are the means ± SD (n = 9). Statistical analysis was performed with one-way ANOVA followed by Tukey’s test. ### *(p* < 0.001) indicates a significant difference from CTR. *** *(p* < 0.001), ** *(p* < 0.01) and * *(p* < 0.05) indicates a significant difference from Hg treatment. MVs: microvesicles; RBC: red blood cell, HT: hydroxytyrosol; Tyr: tyrosol; HA: homovanillic alcohol; FITC: fluorescein isothiocyanate.

**Figure 4:**
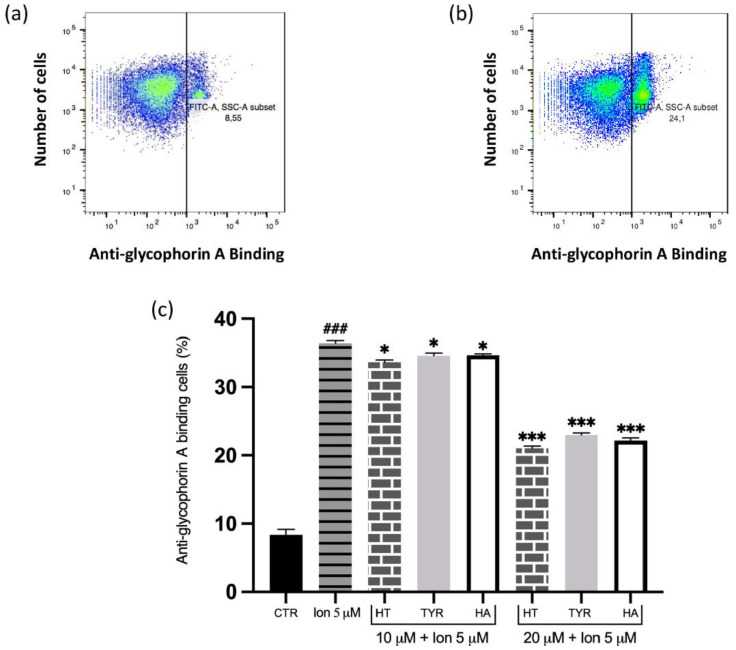
Effect of HT and its analogue on Ca^2+^-induced MVs formation from RBC. Cells were treated with Ca^2+^ ionophore in the presence of increasing concentrations of HT, Tyr and HA and MVs formation was evaluated using FITC anti-human glycophorin A antibody. (**a**) Original histogram of control cells (CTR). (**b**) Original histogram of Ca^2+^ ionophore-treated cells. (**c**) Histogram of phenols effect on Ca^2+^ ionophore treatment. Data are the means ± SD (n = 9). Statistical analysis was performed with one-way ANOVA followed by Tukey’s test. ### *(p* < 0.001) indicates a significant difference from CTR. *** *(p* < 0.001) and * *(p* < 0.05) indicates a significant difference from Ca^2+^ ionophore treatment. MVs: microvesicles; RBC: red blood cell, HT: hydroxytyrosol; Tyr: tyrosol; HA: homovanillic alcohol; FITC: fluorescein isothiocyanate; DFC: dichlofluorescein.

To further elucidate the PS-related membrane alterations, we verified the possible protective effect exerted by HT and its analogues in the prevention of MVs formation induced by Hg and increased intracellular calcium, analyzed by flow cytometry using anti-human anti-glycophorin A FITC antibody. The data reported in Figure 3 and Figure 4 reveal that all the selected phenols prevent MVs formation and that the protective effect parallels that observed for PS exposure. 

**Figure 5:**
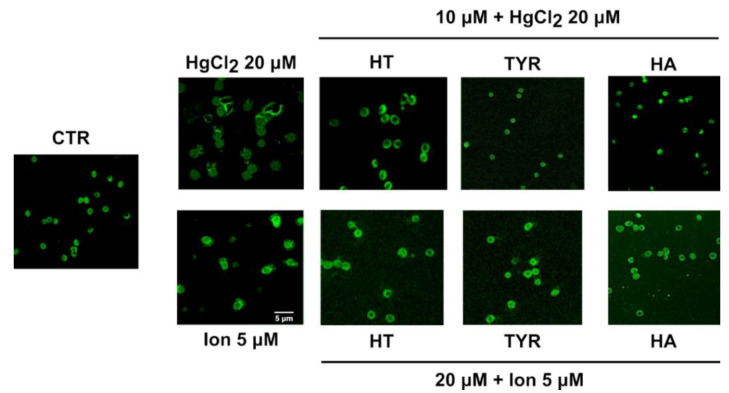
Effect of HT and its analogue on Hg or Ca^2+^-induced MVs release from RBC. Cells were treated with HgCl_2_ and Ca^2+^-ionophore in the presence of HT, Tyr and HA and MVs release were stained with FITC anti-human glycophorin A antibody.

These data have been further supported by confocal fluorescence microscopy (Figure 5). 

**Figure 6:**
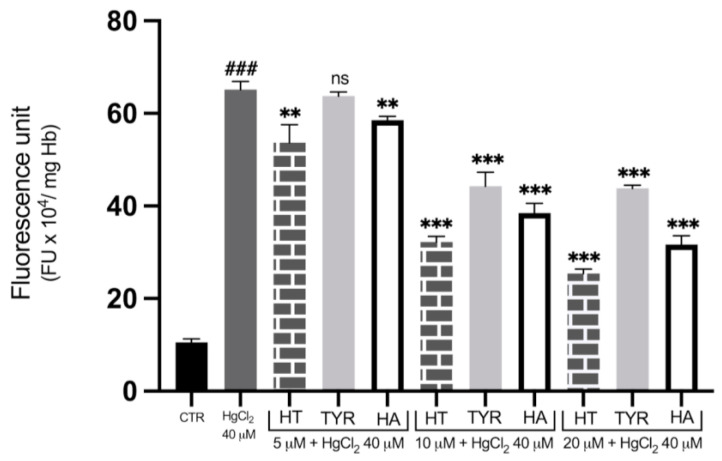
Effect of HT and its analogues on Hg-induced ROS production in RBC. Cells were treated with HgCl_2_ in the presence of increasing concentrations of HT, Tyr and HA and ROS production was evaluated by means of the fluorescent probe DCF. Data are the means ± SD (n = 9). Statistical analysis was performed with one-way ANOVA followed by Tukey’s test. ### (*p* < 0.001) indicates significant difference from control (CTR). *** (*p* < 0.001), ** (*p* < 0.01), indicate a significant difference from Hg treatment. ns (*p* > 0.05) indicates no significant difference from Hg treatment. ROS: Reactive oxygen species; RBC: red blood cell; HT: hydroxytyrosol; Tyr: tyrosol; HA: homovanillic alcohol; DFC: dichlofluorescein.

**Figure 7:**
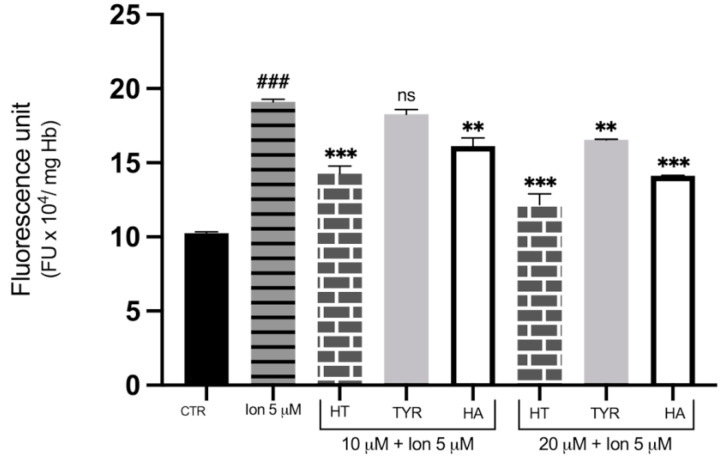
Effect of HT and its analogues on Ca^2+^-induced ROS production in RBC. Cells were treated with Ca^2+^ ionophore in the presence of increasing concentrations of HT, Tyr and HA and ROS production was evaluated by means of the fluorescent probe DCF. Data are the means ± SD (n = 9). Statistical analysis was performed with one-way ANOVA followed by Tukey’s test. ### *(p* < 0.001) indicates a significant difference from control (CTR). *** *(p* < 0.001), ** *(p* < 0.01), indicate a significant difference from Ca^2+^ ionophore treatment. ns (*p >* 0.05) indicates no significant difference from Ca^2+^ ionophore treatment. ROS: Reactive oxygen species; RBC: red blood cell; HT: hydroxytyrosol; Tyr: tyrosol; HA: homovanillic alcohol; DFC: dichlofluorescein.

To investigate the role of OS in Hg-induced cytotoxicity and the possible protective effects of HT, Tyr and HA, the fluorescent dichlofluorescein (DCF) assay was utilized to measure ROS formation. Figure 6 shows that incubation with 40 μM HgCl_2_ causes an increase in DCF fluorescent signal, which is indicative of ROS formation after 4 h of treatment, confirming our previous data that Hg treatment exposes cells to an oxidative microenvironment. To test the efficiency of HT and its analogues in reducing ROS generation, cells were subjected to metal exposure in the presence of increasing concentrations of the three phenols. As shown in Figure 6, HT and HA decrease the fluorescent signal of the metal-exposed RBC, with a significant reduction observed at all concentrations tested, starting at a concentration as low as 5 μM. Tyr also appears protective against Hg-induced ROS formation, although to a lesser extent, with a significant value at 10 μM. Cell treatment with HT and its analogues also results in prevention of ROS generation induced by incubation in the presence of 5 µM of Ca^2 +^ ionophore, the protective effect being less efficient compared with Hg-induced ROS production (Figure 7).

**Figure 8:**
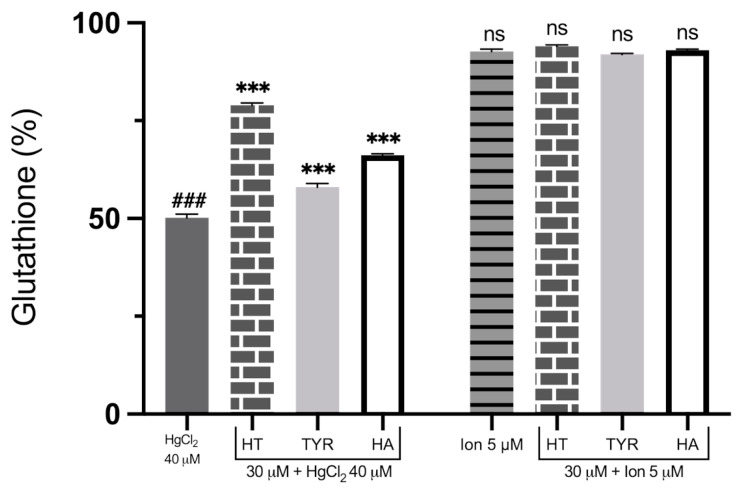
Effect of HT and its analogues on HgCl_2_ or Ca^2+^-induced GSH decrease in RBC. Cells were treated with HgCl_2_ and Ca^2+^ ionophore in the presence of HT, Tyr and HA. Data are the means ± SD (n = 9). Statistical analysis was performed with one-way ANOVA followed by Tukey’s test. ### (*p* < 0.001) indicates a significant difference from control (CTR). *** (*p* < 0.001) indicates a significant difference from Hg treatment. ns (*p >* 0.05) indicates no significant difference from Ca^2+^ ionophore treatment. GSH: glutathione; HT: hydroxytyrosol; Tyr: tyrosol; HA: homovanillic alcohol.

Due to the fundamental role played by GSH depletion in Hg-induced toxicity, linked to the impairment of the antioxidant defense system, we compared the possible protection of HT and its analogues on this specific metabolic alteration induced by HgCl_2_ and Ca^2+^ ionophore. As shown in Figure 8, RBC exposure to the heavy metal for 4 h drastically reduces GSH levels. All phenols are able to partially restore GSH cellular content, Tyr being the least efficient again. Interestingly, intracellular GSH was not significantly reduced when measured upon cell incubation with the Ca^2+^ ionophore.

## 3. Discussion

RBC are the most abundant cells in the bloodstream, characterized by a simplified metabolism and amply utilized as a unique cellular model in biomedical research, due to its peculiar maturation process [7]. In particular, late enucleation is considered an advantage in the investigation of particular aspects of these cells, such as aging [45]. In vitro ageing during the blood bank storage has been also amply investigated [46,47].

Furthermore, RBC are particularly suitable for studies that investigate OS-induced events, due to the high tension of oxygen and the highly toxic free radicals deriving from them [48,49]. Accordingly, these cells are equipped with a powerful antioxidant defence system and significantly contribute to protect from oxidative insult, both other blood cells and the endothelium [22]. However, if RBC reach highly inflamed tissues, such as the endothelium with atherosclerotic lesions, their behaviour shifts from the physiological activity of scavenger to the harmful role of ROS generator, inducing an oxidative microenvironment, thus worsening endothelial dysfunction [50]. Accordingly, RBC have been identified for use as potential markers of OS-related pathologies, including cardiovascular diseases (CVD). Interestingly in this respect, RBC have been proposed as a potential link between diabetes and Alzheimer’s diseases [51]. Finally, we recently reviewed data indicating the rationale for using of RBC as a model for heavy metal-induced endothelial dysfunction, including Hg [35]. In this respect, a link between this highly toxic environmental pollutant and CVD has been proposed, and several authors indicate that Hg human exposure may act as a potential risk factor for these pathologies [52,53].

As far as the mechanisms underlying Hg toxic effects on the cardiovascular system, RBC metabolic as well as morphological changes appear to play a role, increasing the pro-coagulant activity of these cells, particularly related to alteration of cellular membrane asymmetry [21]. The maintenance of phospholipid asymmetry in the plasma membrane, resulting from the coordinated action of flippase and scamblase enzymatic activities [54,55], is essential for RBC physiopathology. In fact, besides its physiological role in erythropoiesis and erythrophagocytosis, PS exposure to the outer membrane leaflet allows the removal of stressed RBC [56]. This membrane modification, however, can induce adverse effects on the cardiovascular system, activating a prothrombotic activity [23]. It is therefore essential to explore tools to protect cells from the possible toxic effects of human exposure to heavy metals. In this regard, we considered worth to verify the potential protective role of nutrition, by testing bioactive compounds normally present in our diet.

The data presented provide experimental evidence for the efficacy of structurally related olive oil phenolic compounds in preventing metabolic and morphological damage to human RBC exposed in vitro to HgCl_2_ treatment and Ca^2+^ ionophore. Our study, in agreement with previous reports [36,57], confirms that Hg and Ca^2+^ induce PS exposure and MVs generation; here, we report that cell treatment with µM concentrations of olive oil phenols reduces the alteration of membrane asymmetry due to PS exposure. In particular, HT and HA show similar protection, while Tyr appears to be less effective. Interestingly, a similar effect was obtained with regard to Ca^2+^ ionophore-induced PS exposure and MVs formation, but the protective effect was observed at higher concentrations of the three phenols, indicating that HT and its analogues also act on non-calcium-dependent mechanisms in Hg toxicity. Accordingly, we report that the removal of calcium from the medium only partially affects the increase in PS exposure induced in RBC by Hg treatment, but HT effect is still observable. In this respect, it is well known that eryptosis is in large part, but not fully, triggered by entry of calcium [58,59]. 

An interesting finding is that all tested compounds are able to maintain cellular thiol homeostasis, counteracting GSH depletion in Hg-exposed RBC. In fact, although a particularly high GSH concentration may partially protect RBC from Hg toxic effects, chronic exposure might affect RBC viability and induce prothrombotic activity, also affecting CVD. In our opinion, the efficacy of the olive oil phenols in preventing Hg-induced modifications in intracellular thiols by restoring the GSH levels may represent a key mechanism underlying the protective effects. It is well known indeed that the simple intracellular thiol modifications can induce apoptosis in nucleated cells, unrelated to ROS generation [60]. Moreover, Mohan et al. reported that the ability of HT to promote the expression of Nrf2, which in turn elevates GSH levels, is crucial in ameliorating the neurotoxic effect of MeHg in IMR-32 neuroblastoma cells [61]. On the contrary, in RBC treated with calcium ionophore, only a minimal reduction in GSH level is observed.

Altogether, data from our study demonstrate that Hg treatment and an increase in intracellular calcium can induce changes in shape, MVs generation, and PS exposure on the RBC membrane, important mediators of RBC procoagulant activation [62]. This suggests that these cells represent key cellular targets of Hg toxicity, thus contributing to the cardiovascular dysfunction associated with human exposure to this heavy metal. In our opinion, the reported findings are particularly promising and clinically important and point to RBC as a target for therapeutic strategies. This aspect deserves further clinical and experimental investigation.

Finally, our findings strengthen the nutritional relevance of olive oil bioactive compounds to the claimed health-promoting effects of this component of the Mediterranean Diet. In this respect, antioxidant polyphenols are believed to play a major role in the positive correlation between adherence to the Mediterranean Dietary Habit and a low incidence of CVD [63,64]. In recent years, nutritional research as well as in vitro studies have mainly focused on the effects of HT in the progression of atherosclerosis [65]. Beside its inflammatory properties, this phenol reduces the expression of adhesion molecules [66,67], a key mechanism implicated in plaque formation. Furthermore, HT inhibits in vitro LDL oxidation and counteracts the OS-induced endothelial dysfunction [68]. The reported data expand upon the HT known beneficial effects of olive oil, particularly related to human exposure to heavy metals, indicating that prevention of metal toxicity should be regarded as an additional mechanism responsible for the health-promoting potential of olive oil intake on the cardiovascular system. In this regard, an important finding is that the in vivo HT metabolite still retains the biological activities of its dietary precursor. Our attention was also directed towards Tyr, which exerts significant protection in spite of its weak scavenging activity [37]. This phenol, which lacks the *o*-diphenolic structure, is unable to counteract the OS-induced cytotoxicity [69], indicating that additional mechanisms are involved in the protective effect observed in our experimental model. However, although Tyr is not as efficient as other antioxidants found in olive oil, our data suggest that it may contribute to the overall positive effect of olive oil on human health. 

The beneficial properties of olive oil bioactive components provide biochemical bases for nutritional strategies in the prevention of pathologies related to Hg exposure, by using both functional foods and nutraceutical preparations. Accordingly, the Mediterranean Dietary Habit decreases prothrombotic MVs release in asymptomatic individuals at high cardiovascular risk [70]. At least in theory, olive oil phenols may be similarly effective in the prevention of endothelial dysfunction-related erythroid and non-erythroid human pathologies [71]. In this respect, RBC-altered adhesiveness appears to play a role in complications such as thrombosis in polycythemia vera and splenic sequestration in hereditary spherocytosis. In addition, RBC adhesion to the vascular endothelium might be involved in the occurrence of vaso-occlusive crisis in sickle cell disease. Unexpectedly, recent data suggest that PS-mediated abnormal RBC adhesion might be involved in the pathophysiology of non-erythroid disorders, such as central retinal vein occlusion and Gaucher disease, which share common clinical manifestations, including thrombotic events [72,73].

## 4. Materials and Methods

### 4.1. Chemicals and Solutions

4-Bromo-calcium Ionophore A23187; DCFH-DA (2′,7′-dichlorodihydrofluorescein diacetate), DTNB (5,5-dithiobis (2-nitrobenzoic acid), or Ellman’s reagent), PBS (phosphate-buffered saline), HgCl_2_, HT, Tyr and HA were from Sigma Chemical Co. (St. Louis, MO, USA), Annexin V-fluorescein isothiocyanate (V-FITC) Apoptosis Detection Kit (556547, BD Pharmigen, Franklin Lakes, NJ, USA) and Anti Glicophorin A antibody (FITC) were purchased from antibodies-online.com (ABIN6253946, antibodies-online GmbH, Aachen, Germany. https://www.antibodies-online.com/antibody/6253946/anti-Glycophorin+A+GYPA+antibody+FITC/ accessed on 12 April 2022). HT, Tyr and HA were prepared in dimethyl sulfoxide (DMSO) and diluted from 10 mM or 100 mM stock Solution. The resulting solutions were preventively tested upon RBC at their final concentrations to exclude any damage.

### 4.2. Preparation of RBC and Treatment with HgCl_2_ and Ca^2+^-Ionophore 

Whole blood was obtained with informed consent from healthy volunteers at Campania University “Luigi Vanvitelli” (Naples, Italy). It was collected in heparinized tubes and centrifuged at 2000× *g* for 10 min at 4 °C. After removal of the buffy coat, the RBC fraction was washed twice with isotonic saline solution (0.9% NaCl) and resuspended in Krebs solution containing (mM) NaCl 125, KCl 4, MgSO_4_ 1, Hepes 32, CaCl_2_ 1, glucose 2.8; pH 7.4 to obtain a 10% (*v/v*) hematocrit (or 0.5 and 3% according to experienced needs). RBC were incubated at 37 °C for 4 or 24 h with HgCl_2_ (5–40 μM) and increasing concentrations of HT, HA and Tyr (1–30 μM). In order to estimate the different impact of Hg and Ca^2+^, RBC were exposed for 1 h to a combination of Ca^2+^-ionophore (5 μM) and different concentrations of the selected phenolic compounds.

### 4.3. Detection of Annexin-V-Binding Cells

After incubation under the respective experimental condition (24 h for Hg treatment), RBC (1 × 10^−6^ for each condition) were resuspended in 600 μL of 1× binding buffer and incubated in the dark for 15 min at room temperature, with 5 μL of annexin V Apoptosis detection kit. The assessment of fluorescence was performed with BD Accuri C6 and data were analyzed by FlowJo V10 software (https://www.flowjo.com/solutions/flowjo accessed on 12 April 2022); 20,000 events were recorded for each sample.

### 4.4. Quantification Assay of MVs by Flow Cytometry 

MVs obtained after HgCl_2_ (24 h treatment) and Ca^2+^-ionophore-stimulation were counted by flow cytometry [74]. Briefly, 95 μL of samples were mixed with 5 μL of anti-human anti glycophorin A FITC-antibody and incubated for 20 min at room temperature (RT) on a roller. Before analysis, 80 μL of labelled MVs were dissolved in 400 μL of 0.9% NaCl and transferred to 1.5 mL tubes. MVs were quantified by flow cytometry, as previously described.

### 4.5. Confocal Microscope Analysis

RBC were treated with HgCl_2_ and Ca^2+^ ionophore in the presence or the absence of HT, TYR and HA, as described above. After incubation (24 h for Hg treatment), RBC were washed twice with phosphate-buffered saline pH 7.4 (PBS) and counted in a Burker chamber. The confocal laser scanning microscope analyses were performed according to Nguyen [74], with few modifications. In brief, the cells were then fixed with 2% formaldehyde for 1 h at 4 °C, then washed several times and incubated with anti-human anti glycophorin A FITC antibody for 30 min at 4 °C in the dark. Afterwards, the samples were placed on glass slides and air-dried for 1 h. The slides were dipped quickly, and gently washed stepwise with ethanol from 50% to 75%, 90%, and then 100% for dehydration. Finally, cells were fixed in 2% formaldehyde and washed three times with PBS. For confocal laser scanning microscope imaging, several randomly selected frames from each sample were captured for morphological observation and statistical strength. Excitation and emission filters were set at 488 nm and 550–600 nm, respectively.

### 4.6. ROS Determination

ROS generation was determined using the DCF assay, according to Tagliafierro et al. [75]. Using this method, 250 µL of intact RBC (hematocrit 10%) were incubated with DCFH-DA at a final concentration of 10 µM for 15 min at 37 °C. After centrifuging at room temperature at 1200× *g* for 5 min, the supernatant was removed, and the hematocrit was re-adjusted to 10% with Krebs solution. RBC were then treated concurrently with HgCl_2_, Ca^2+^-ionophore and selected phenolic compounds in the dark (4 h for Hg treatment). After the incubation, 20 µL of RBC were diluted in 2 mL of water, and the fluorescence intensity of the oxidized derivative DCF was recorded (_exc502; _em520). The results were expressed as fluorescence intensity/mg of hemoglobin. Fluorescence measurements were performed on a Perkin Elmer Life Sciences LS 55 spectrofluorimeter.

### 4.7. Assay for Reduced GSH

By reaction with DTNB, the intracellular GSH content was assessed, according to Tortora et al. [76]. After incubation (4 h for Hg treatment), samples (0.25 mL) were centrifuged for 5 min at 800× *g*. After removal of supernatants, RBC were lysed using 0.6 mL of cold water and proteins were then precipitated by the addition of 0.6 mL of a cold metaphosphoric acid solution (1.67 g metaphosphoric acid, 0.2 g EDTA, and 30 g NaCl in 100 mL of water). The samples were placed at 4 °C for 5 min, and then the precipitated proteins were removed by centrifugation at 18,000× *g* for 10 min. Finally, 0.45 mL of supernatant were mixed with an equal volume of 0.3 M Na_2_HPO_4_. To determine the reduced GSH, 0.1 mL of a DNTB solution (20 mg DTNB plus 1% of sodium citrate in 100 mL of water) was added to the solution. After 10 min of incubation at room temperature, the absorbance of the samples was measured at a wavelength of 412 nm.

### 4.8. Statistical Analyses

Data evaluations were expressed as means ± S.D. of 3 independent experiments performed in triplicate with RBC from different donors. The significance of differences was determined by one-way ANOVA followed by a post Tukey’s multiple comparisons test. GraphPad Prism 9.1 was utilized for statistical analysis.

## Data Availability

Not applicable.

## Abbreviations

CVD	Cardiovascular Diseases
DCF	Dichlofluorescein
DCFH-DA	2′,7′-dichlorodihydrofluorescein diacetate
DMSO	Dimethyl sulfoxide
DNTB	5,5-dithiobis 2-nitrobenzoic acid
GSH	Glutathione
HA	Homovanillic alcohol
Hg	Mercury
HT	Hydroxytyrosol
MVs	Microvesicles
NO	Nitric oxide
OS	Oxidative stress
PBS	Phosphate-buffered saline
PS	Phosphatidylserine
RBC	Erythrocytes
ROS	Reactive Oxygen Species
Tyr	Tyrosol
